# A review of deep learning for brain tumor analysis in MRI

**DOI:** 10.1038/s41698-024-00789-2

**Published:** 2025-01-03

**Authors:** Felix J. Dorfner, Jay B. Patel, Jayashree Kalpathy-Cramer, Elizabeth R. Gerstner, Christopher P. Bridge

**Affiliations:** 1 Athinoula A. Martinos Center for Biomedical Imaging, 149 13th St, Charlestown, MA 02129 USA; 2 University of Colorado School of Medicine, Anschutz Medical Campus, Aurora, CO 80045 USA; 3 Massachusetts General Hospital Cancer Center, Boston, MA 02114 USA

**Keywords:** CNS cancer, Mathematics and computing

## Abstract

Recent progress in deep learning (DL) is producing a new generation of tools across numerous clinical applications. Within the analysis of brain tumors in magnetic resonance imaging, DL finds applications in tumor segmentation, quantification, and classification. It facilitates objective and reproducible measurements crucial for diagnosis, treatment planning, and disease monitoring. Furthermore, it holds the potential to pave the way for personalized medicine through the prediction of tumor type, grade, genetic mutations, and patient survival outcomes. In this review, we explore the transformative potential of DL for brain tumor care and discuss existing applications, limitations, and future directions and opportunities.

## Introduction

Deep learning (DL), a form of artificial intelligence (AI), is rapidly transforming various fields, demonstrating remarkable success in tackling complex challenges, such as image recognition and natural language processing. These capabilities of DL have also found applications within medicine, with DL models having demonstrated effectiveness on tasks such as medical text summarization, prediction of future lung cancer risk, prediction of SARS-CoV-2 infectivity and variant evolution, identification of new antibiotics, and assessment of mammography for breast cancer^1–5^. In this review, we specifically focus on the applications of DL to brain tumor image analysis where there have been several important advances as well.

Brain tumors are the most common solid tumors in children and adolescents. Annually, more than 88,000 adults and 5500 children are diagnosed with brain tumors in the United States alone. These tumors have very high mortality, with a 5-year relative survival rate following diagnosis of a malignant brain or other CNS tumor of only 35.6% in adults^6^.

According to the 5th edition of the WHO classification, tumors of the central nervous system (CNS) are classified into different tumor grades based on histological, immunohistochemical, and molecular features^7^. Diffuse gliomas (which include Astrocytomas, Oligodendrogliomas, and Ependymomas) are the most common type of primary malignant CNS tumor in adults, making up about 25% of all such cases^6^. The most common primary non-malignant brain tumors in adults are meningiomas. And the overall most common type of brain tumor is brain metastases, as about 20% of all patients with cancer will develop brain metastases during the course of their treatment^8^.

Within neuro-oncology, the need for DL-powered solutions is directly related to the complexities associated with brain tumors. Brain tumors exhibit substantial heterogeneity in their presentation and require diverse therapeutic approaches. Analyzing these tumors accurately and efficiently is crucial for optimizing patient care. DL can play a pivotal role in this regard in one of two broad capacities. The first set of applications of DL models is on tasks that are very time-consuming for human experts within the existing clinical workflow, such as the creation of 3D segmentation masks for tumor quantification. However, beyond this, DL models have been shown to be capable of extracting insights beyond human capabilities, such as the prediction of important genomic biomarkers based on MRI alone^9^. As these capacities mature and develop, DL may help shape the workflows of tomorrow.

This review explores the transformative impact of DL on brain tumor analysis, focusing on its applications in two broad areas: segmentation and classification. We discuss how DL models are enabling automated and accurate tumor segmentation from medical images, facilitating objective and reproducible measurements crucial for diagnosis, treatment planning, and disease monitoring.

We also provide an outlook on current innovations for medical DL models. Namely, we discuss the growing role of foundation models, which are models trained on massive datasets of diverse data types, in the field of medicine. We anticipate that these models will greatly enhance the accuracy of DL-based brain tumor analysis and enable researchers to extend it toward tumor types for which analysis was previously unfeasible due to the limited amount of training data available.

## Deep learning methods for MRI analysis

As a general term, AI refers to the development of computer systems capable of performing tasks that typically require human intelligence, such as visual perception, decision-making, and learning.

The first generation of AI applications for MRI data included *radiomics*-based approaches. Here, predefined sets of features—quantifying image characteristics such as intensity, contrast, shape or texture—were extracted from the image and then passed into a classical machine learning model, such as random forests or support vector machines, to make predictions^10,11^. Their application to brain tumors has been extensively covered by previous reviews^12^. By contrast, DL models use artificial neural networks to learn complex patterns and relationships *directly* from the data, during a process called *training*. During training, a model iteratively improves by adjusting the internal model parameters to minimize the difference between its predictions and the known ground-truth labels. By analyzing vast amounts of information, these deep learning models can identify subtle features and make predictions that may not be readily apparent to human observers. Since features in DL models are optimized directly for a particular task, they are often able to make better predictions than the previous generations of radiomics-based approaches.

**Fig. 1:**
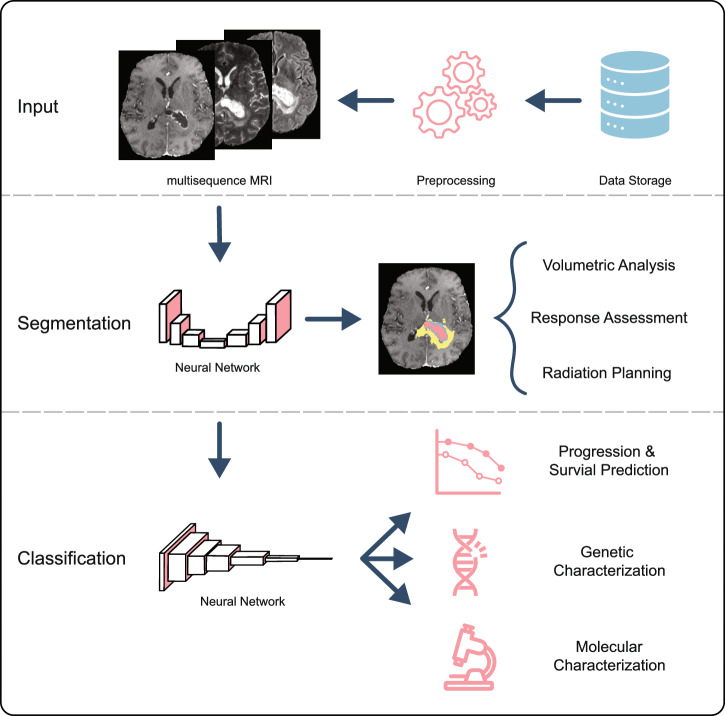
Examples of deep-learning-based workflows for MRI segmentation and classification. For the segmentation task, the CNN receives an input image, often consisting of multiple sequences, and outputs a segmentation map according to the given task, such as segmenting a tumor. For the classification task, the model receives the input image and outputs a classification into two or more classes.

The majority of modern deep learning-based architectures for image analysis are based on convolutional neural networks (CNN)^13^. The core component of a convolutional neural network is a convolutional layer, which moves a learned filter across the image in a “sliding-window” fashion. This approach uses fewer parameters compared to a simple “fully-connected” neural network and is able to recognize the learned patterns regardless of their location in the image. Each convolutional layer in the network allows for increasing abstraction and identification of more complex features. Within the scope of image analysis, tasks can be broadly split into two groups: image segmentation (i.e. delineating salient/relevant regions on the image) and image classification (i.e. categorizing the image from a set of pre-determined classes). As illustrated in Figure 1, these deep learning models have shown state-of-the-art performance across a wide range of neuro-oncology tasks, including but not limited to tumor segmentation, prediction of mutation status for gliomas, and prediction of treatment response^14–16^.

Since brain MRIs are volumetric images, it is natural to employ convolutions along all three dimensions, giving rise to 3D CNNs^17^. While this is the more common approach for analyzing brain MRI and allows the model to fully consider volumetric information when making predictions, there are some drawbacks compared to the 2D CNNs that are more common in other application domains. Firstly, 3D networks typically have more parameters, leading to higher computational demands and making them more challenging to optimize especially on small datasets. Additionally, powerful 2D networks pre-trained on large datasets of 2D images are readily available to use as the basis of new models via transfer learning^18^, but a limited choice of 3D architectures is available in this way. Finally, since thick slice MRI data is highly anisotropic, it is not necessarily optimal to treat all three spatial dimensions equivalently within the model. Thus some authors use 2D CNNs applied to each 3D slice of the MRI, potentially with immediately adjacent slices included as additional channels in a so-called “2.5D” architecture. Mixed results suggest that the optimal choice of architecture may be dependent on application and training dataset^19–21^.

## Segmentation and quantification

Perhaps the most well-studied application of DL within neuro-oncology is that of tumor segmentation. Segmentation is the process of delineating tumor regions (or sub-regions) within an image and is a key step in tumor quantification, response assessment, and treatment planning, as well as a preliminary step for further analyses of different tumor regions (see the section “Classification”).

### Public datasets and benchmarks

The proliferation of work on segmentation has been made possible thanks to the wide availability of relevant publicly accessible data. Publicly available datasets allow researchers around the world to train DL models on multi-institutional, high-quality datasets and thus serve as a benchmark for comparing models. The largest public datasets of brain tumor MRI images are listed in Tables 1–3.

**Table 1:** Overview of public datasets for MRI studies of brain tumors

Public Dataset	Data Publisher	No. of Cases/Patients	Tumor Type
BraTS 2021^a^^93^	RSNA-ASNR-MICCAI	2000 patients	Adult diffuse glioma
BraTS-Africa 2023^23^	MICCAI-CAMERA- Lacuna Fund	95 cases	Adult diffuse glioma
BraTS-PEDs 2023^22^	CBTN-CONNECT- DIPGR-ASNR-MICCAI	228 patients	Pediatric high-grade glioma
BraTS Meningioma 2024^24^	RSNA-ASNR-MICCAI	1650 cases	Meningioma
BraTS-METS 2023^b^^30^	RSNA-ASNR-MICCAI	328 cases	Brain metastasis
NYUMets^28^	New York University	1429 patients	Brain metastasis
UPenn-GBM^132^	University of Pennsylvania	630 patients	Glioblastoma
UCSF-BMSR^26^	University of California San Francisco	412 patients	Brain metastasis
TCGA-GBM^133^	TCGA Glioma Phenotype Research Group	262 patients	Glioblastoma
Figshare Dataset^134^	Southern Medical University, Guangzhou, China	233 patients	Glioma, meningioma, pituitary
GLIS-RT Dataset^82^	Massachusetts General Hospital	230 patients	Glioblastoma, astrocytoma, low-grade glioma
Pretreat-MetsToBrain-Masks^135^	Yale School of Medicine	200 patients	Brain metastasis
TCGA-LGG^136^	TCGA Glioma Phenotype Research Group	199 patients	Low-grade glioma
BrainMetShare^29^	Stanford University	156 patients	Brain metastasis
CPTAC-GBM^137^	National Cancer Institute’s Clinical Proteomic Tumor Analysis Consortium	99 patients	Glioblastoma
MOLAB^25^	University of Castilla-La Mancha	75 patients	Brain metastasis
Brain-TR-Gamma-Knife^27^	University of Mississippi	47 patients	Brain metastasis
IVY GAP^138^	Allen Institute for Brain Science	39 patients	Glioblastoma

^a^ Contains a subset of studies from the TCGA-GBM, TCGA-LGG, IVY GAP, and CPTAC-GBM Datasets.

^b^Subset of studies from the NYUMets, UCSF-BMSR, Pretreat-MetsToBrain-Masks, and BrainMetsShare Datasets.

**Table 2:** Overview of model architectures, training data, and metrics results from selected papers

Rerence	Model architecture name	Training data used	Test set results (Dice)
Myronenko et al.^34^	Asymmetrical U-Net	BraTS 2018	WT: 88.39, TC: 81.54, ET: 76.64
Jiang et al.^35^	Two-Stage Cascaded U-Net	BraTS 2019	WT: 88.80, TC: 83.70, ET: 83.27
Isensee et al.^33^	nnU-Net (no new-Net)	BraTS 2020	WT: 88.95, TC: 85.06, ET: 82.03
Luu and Park^36^	modified nnU-Net	BraTS 2021	WT: 92.75, TC: 87.81, ET: 84.51
Zeineldin et al.^14^	Ensemble: DeepSeg, nnU-Net, and DeepSCAN	BraTS 2022	WT: 92.94, TC: 87.88, ET: 88.03

*WT* whole tumor, *TC* tumor core, *ET* enhancing tumor.

**Table 3:** Overview of model architectures, training data, and metrics results from selected papers for classification tasks

Reference	Dataset	Input	No. Patients	Task	Metric (result)
*Recurrence vs. radiation necrosis*					
Gao et al.^87^	Private	T1, T1-c, T2	146	Recurrence vs. radiation necrosis	AUC: 0.915
Lee et al. (2020)^88^	Private	T1, T1-c, T2, T2-FSE, FLAIR, ADC	46	Recurrence vs. radiation necrosis	AUC: 0.81
*Survival prediction*					
McKinley et al. (2020)^89^	BraTS 2020	T1, T1-c, T2, FLAIR	587	Overall survival (>15, 15–10, <10 months)	Accuracy: 0.617
Yan et al. (2023)^90^	BraTS 2019	T1, T1-c, T2, FLAIR	205	Overall survival (>15, 15–10, <10 months)	Accuracy: 0.548
*IDH status prediction*					
Chang et al. (2018)^9^	Private/TCIA	T1, T1-c, T2, FLAIR	291	IDH status prediction	AUC: 0.95
Choi et al. (2020)^15^	Private/TCIA	T1-c, T2, FLAIR	1166	IDH status prediction	AUC: 0.86–0.96
*Molecular biomarker prediction*					
Tak et al. (2024)^91^	Private/CBTN	T2	326	BRAF mutational status prediction	AUC: 0.73–0.82
Calabrese et al. (2020)^92^	Private	T1, T1-c, T2, FLAIR	199	9 Molecular biomarkers prediction	AUC: 0.55–0.97
Chen et al. (2020)^94^	TCIA	T1-c/FLAIR	106	MGMT methylation status prediction	AUC: 0.828–0.897
Yogananda et al. (2021)^95^	TCIA	T2	247	MGMT methylation status prediction	AUC: 0.93
Chen et al. (2022)^96^	Private	T1, T1-c, T2, ADC	111	MGMT methylation status prediction	AUC: 0.9
Saeed et al. (2022)^97^	BraTS 2021	T1, T1-c, T2, FLAIR	585	MGMT methylation status prediction	AUC: 0.54–0.64
Robinet et al. (2023)^98^	BraTS 2021/Private	T1, T1-c, T2, FLAIR	672	MGMT methylation status prediction	AUC: 0.60–0.65

Some of the most widely used public datasets come from the Brain Tumor Segmentation (BraTS) challenge, hosted annually by the Medical Image Computing and Computer-Assisted Intervention (MICCAI) Society since 2012. Initially focused solely on the segmentation of gliomas, BraTS has expanded over the years to include other CNS tumors. In 2022, BraTS introduced a pediatric dataset and a dataset of adult-type diffuse glioma of underrepresented patients (BraTS-Africa)^22,23^, and in 2023, challenges for the segmentation of brain metastases and meningiomas^22,24^ were added. Due to the scope, size, and accessibility of these datasets, BraTS has become an important benchmark for state-of-the-art brain tumor segmentation.

One challenge is that public datasets can greatly vary in quality and content. For example, consider datasets available for brain metastases segmentation projects. While some provide just a single imaging sequence (e.g. MOLAB^25^), others may provide multiple sequences (e.g. UCSF-BMSR^26^). Some may provide the raw images (e.g. Brain-TR-GammaKnife^27^), whereas others may provide only pre-processed versions (e.g. NYUMets^28^). Differences in annotations may also exist, with some datasets providing binarized (tumor vs. no tumor) labels (e.g. BrainMetShare^29^) and others providing multi-class (contrast-enhancing tumor, necrosis, and peritumoral edema) labels (e.g. BraTS-METS^30^). As such, every dataset is composed of images and annotations tailored towards a specific endpoint, which can create non-trivial problems for combining datasets from different sources to solve a single task such as segmentation.

### Segmentation approaches and architectures

As the BraTS challenge is an important benchmark for the performance of segmentation models, examining the winning architectures of the past challenges provides an overview of the evolution of medical image segmentation architectures.

One fundamental segmentation model is the U-Net, which was first introduced by Ronneberger et al. in 2015^31^ and has been the basis of the winning segmentation models in BraTS since then as well as becoming ubiquitous in other medical image segmentation tasks such as segmenting intracranial metastases^32^ and many other biological structures^33^. The U-Net is composed of two main structures: the contracting path gradually downsamples the image and extracts features at lower spatial resolution and a higher semantic level, and then the expanding path re-combines these features to create the segmentation mask by gradually upsampling the image again to the input resolution. Skip connections between layers in the contracting path and the expanding path are used to preserve detailed information lost during downsampling, ensuring fine detail in the output segmentation mask.

Various modifications to the standard U-Net have since been proposed to improve performance. A crucial early improvement was the introduction of the Dice loss by Milletari et al.^17^. The Dice loss function, based on the similar Dice similarity coefficient (DSC) measures the overlap between the predicted foreground region and the ground truth foreground region regardless of the size of the foreground region. As a result, it is suitable for segmentation problems in which there exists a large class imbalance between the foreground and background classes, and/or in which foreground sizes vary considerably between different samples in the dataset, unlike the common cross-entropy loss. In 2018, Myronenko placed first in the BraTS segmentation challenge by utilizing an asymmetrical U-Net with residual blocks, which contain shortcuts within the network that help preserve information and improve learning during training^34^. In 2019, the winning architecture was a cascade of two similar U-Nets, where the additional second model was used to refine the coarse segmentation maps generated from the first^35^. In 2020, Isensee et al. proposed No-New-Net (nnU-Net), which built a framework around a standard U-Net architecture and automated most of the deep learning pipeline including image pre-processing, model adaptation, hyperparameter tuning, and an ensembling strategy, leading to improved performance and consistency^33^. Ensembling is a technique in which different models are trained to perform the same task and their individual outputs are combined into one final prediction. The segmentation winner in 2021 improved on this nnU-Net solution by incorporating an asymmetric contracting path to achieve a better balance of resources between a more powerful contracting path and a simpler expanding path, group normalization for more robust training with the small batch sizes necessary when using large 3D images, and an axial attention decoder focus to model attention on relevant parts of the input image^36^. This improved architecture was ensembled together with DeepSeg and DeepScan to create an even stronger solution for the 2022 segmentation challenge^14,37,38^.

Object detection networks, which output coarse bounding box predictions rather than detailed voxel-wise classification, may be used as an alternative to segmentation. This may be more appropriate where the detection of lesions is more important than quantification and measurement, and has the key advantage that they can be trained using simple bounding box annotations, which are much quicker to perform than segmentation masks. For example, Zhang et al.^39^ use the Faster R-CNN architecture^40^ for the detection of brain metastases. Furthermore, in some approaches such as DeSeg^41^, object detection outputs can be used to screen for initial locations that can be fed to further models to perform detailed segmentation.

### Multi-sequences and missing sequences

Typically, CNNs for MRI image analysis are trained on a specific sequence or set of sequences. Similar to a trained radiologist, the model processes different sequences of a study to make its decision, as these sequences highlight different aspects of the tumor. However, this multi-sequence training can be a limitation at inference time if an imaging study does not have all the required sequences. Therefore, several approaches have been developed to deal with missing sequences for MRI imaging studies in order to increase the applicability of trained models.

One approach includes network architectures that are designed to accommodate variable input sequences and have been explicitly trained for this task. One example of this is the Hetero-modal Image Segmentation approach by Havaei et al.^42^. Here, the model processes each sequence input independently to create a high-level representation of each image. These representations are then combined via simple operators (such as the mean or standard deviation) in such a way as to ensure that an arbitrary number of sequences can be provided to the model at test time. The combined representation is then passed to further layers of the network for segmentation. Using a different approach, Feng et al. directly trained a model to handle missing data by randomly replacing input sequences with empty images. This allowed the model to adapt to missing sequences and still perform the segmentation if one or more required sequences were missing^43^.

A second approach generates missing sequences so that they can then be used in models that expect a fixed set of input sequences. State-of-the-art methods mostly use generative adversarial networks (GAN) to generate the missing target sequence from the available input sequences. Two important differences between sequence generation models are which sequences are required to generate the missing sequence and whether the input images need to be spatially aligned. One approach used this idea to generate T1-c and Double inversion recovery (DIR) images from three common input sequences (T1, T2, FLAIR)^44^. In addition to GANs, diffusion-based models, which gradually remove noise from a random image to generate realistic data, have also been proposed for the generation of missing MRI sequences^45^.

### Site generalization

It has been shown that DL model performance can suffer significantly if applied to data from different sites, and MRI is particularly vulnerable to this issue due to its flexibility and differences across scanners and protocols^46^. One approach to address this is harmonization, where techniques like StarGAN and CycleGAN standardize image appearance across sites to improve model robustness^47–50^. StarGAN and CycleGAN are both generative adversarial networks (GAN) that are trained by having the image generation model ("generator”) compete with a model that tries to identify which images are real and which are synthetic ("discriminator”). As training in this way progresses, the generator generates increasingly realistic images. Another strategy is to apply extensive augmentation during training, adding diverse artifacts to expose the model to a range of imaging conditions. Methods such as SynthSeg, SynthMorph, and SynthStrip demonstrate that this approach can improve generalization across sites^51–53^ for multiple tasks.

### Pediatric brain tumors

Pediatric brain tumors represent the most common cause of cancer-related mortality in children^22^. Although some parallels exist with adult brain tumors, pediatric tumors often exhibit distinct imaging characteristics and clinical presentations. For instance, adult glioblastomas (GBMs) and pediatric DMGs are both high-grade gliomas associated with poor prognoses; however, their incidence rates and typical locations differ. GBMs, with an incidence of ~3 per 100,000, predominantly affect older adults and are frequently found in the frontal and/or temporal lobes, whereas DMGs are considerably rarer and typically arise in the pons of children aged 5–10 years. Furthermore, characteristic imaging features like post-gadolinium enhancement and necrosis, common in GBMs, are less consistently observed in DMGs, particularly at initial diagnosis^54,55^. Consequently, specialized imaging tools are critical for characterizing and assessing these pediatric tumors, deep learning models that were developed adult brain tumors can not just be applied to pediatric brain tumors, due to the differences between tumor presentations. As a result, separate deep-learning models need to be developed for most applications of segmenting and analyzing pediatric brain tumors. The BRATS challenge included a dataset of pediatric tumors for the first time in 2023. This dataset contains MRI sequences (T1, T1-c, T2, FLAIR) for 228 patients with pediatric high-grade gliomas. The winning team here achieved a mean dice score of 0.65 for ET, 0.81 TC, and 0.83 for segmenting the whole tumor^56^.

### Segmentation evaluation metrics

There are a variety of methods and metrics for evaluating the performance of deep-learning models. Choosing the appropriate metric for a given problem is crucial to ensure that it accurately captures the clinically relevant aspects of the task. A recent study proposed a framework called Metrics Reloaded, which provides a tool to guide researchers through the process of choosing the right validation metrics for their DL model^57^.

The two most important metrics for brain tumor segmentation are the Dice score and the Hausdorff distance (HD). The Dice score measures the overlap between the predicted segmentation and the ground truth segmentation. It ranges from 0 (no overlap) to 1 (perfect overlap) and provides an intuitive assessment of segmentation accuracy that measures only the degree of overlap between the prediction and ground truth, regardless of the absolute size of the region. The Hausdorff distance, on the other hand, quantifies the maximum distance between any point on the predicted segmentation boundary and the nearest point on the ground truth boundary. As a distance metric, the HD ranges from 0 to infinity. To mitigate the effect of outliers, the 95th percentile HD is often used. While these metrics capture the agreement between the model prediction and the ground truth, it has been shown that the scores may have a poor correlation with clinician perception of segmentation quality^58^. Furthermore, currently, DL-based segmentation is often corrected by clinicians to ensure quality. Here, clinicians may prefer certain patterns in the model’s segmentation behavior, for example over-segmentation rather than under-segmentation, as it may be easier to correct. These practical preferences are not captured by popular metrics of segmentation performance but may play an important role in clinical adoption^59^.

1 $${\rm{Dice}}\, {\rm{Similarity}}\, {\rm{Coefficient}}\, {({\rm{DSC}})}\,=\frac{2\,| {GT}\cap {MS}| }{| {GT}| +| {MS}| }$$

$$\begin{array}{l}{GT}={\rm{Ground}}\, {\rm{Truth}}\, {\rm{Surface}}\\ {MS}={\rm{Masked}}\, {\rm{Segmentation}}\end{array}$$

2 $${\rm{Average}}\, {\rm{Hausdorff}}\, {\rm{Distance}}\, {({\rm{AHD}})}\,=\max \left(d\left({S}_{{\rm{GT}}},{S}_{{\rm{MS}}}\right),\,d\left({S}_{{\rm{MS}}},{S}_{{\rm{GT}}}\right)\right)$$

$$\begin{array}{l}{S}_{{\rm{GT}}}={\rm{Ground}}\, {\rm{Truth}}\, {\rm{Surface}}\\ {S}_{{\rm{MS}}}={\rm{Masked}}\, {\rm{Segmentation}}\, {\rm{Surface}}\\ d={\rm{Distance}}\, {\rm{Function}}\end{array}$$

3 $${\rm{Accuracy}}=\frac{{\rm{TP}}+{\rm{TN}}}{{\rm{TP}}+{\rm{TN}}+{\rm{FP}}+{\rm{FN}}}$$

$$\begin{array}{l}{TP}={\rm{True}}\, {\rm{Positives}}\\ {TN}={\rm{True}}\, {\rm{Negatives}}\\ {FP}={\rm {False}}\, {\rm{Positives}}\\ {FN}={\rm {False}}\, {\rm{Negatives}}\end{array}$$

4 $${\rm{AUROC}}=\mathop{\int}\nolimits_{0}^{1}{\rm{TPR}}(t)\,d{\rm{FPR}}(t)$$

$$\begin{array}{l}{{TPR}}={\rm{True}}\, {\rm{Positive}}\, {\rm{Rate}}\\ {{FPR}}={\rm {False}}\, {\rm{Positive}}\, {\rm{Rate}}\end{array}$$

### Uncertainty

Another important aspect of the clinical application of deep-learning-based segmentation models is the quantification of segmentation uncertainty. This approach can help clinicians when they are manually revising model segmentations, as areas marked as uncertain are most likely to contain mistakes. The implementation of uncertainty can also help to build trust in DL models. As part of the BraTS challenge in 2020, a metric to compare the performance of uncertainty measures was introduced, and a variety of solutions to the problem were submitted by participants^60^.

McKinley et al. proposed a novel loss function (a function that measures the difference between the model prediction and the ground-truth) for the task of uncertainty estimation in brain tumor segmentation^38^. In their approach, the model outputted two probabilities for each voxel in the input image; one that the predicted label was correctly identifying the ground truth label and one that the predicted label did not correspond to the ground-truth label. These two outputs were used during model training to jointly optimize tumor segmentation and uncertainty estimation. Other groups have directly utilized the scores that the model outputs for each possible label in the segmentation mask to derive uncertainty scores^61,62^. These types of approaches can be further improved by combining models into an ensemble, leveraging the strengths of each individual model for a more accurate and robust outcome.

Other methods of quantifying uncertainty are based on the idea of creating a distribution of outputs for a single input image and using statistics of that distribution to estimate uncertainty. In Monte Carlo dropout, a fraction of the nodes in the network are randomly deactivated ("dropped out”) and inference is performed a number of times, producing a distribution of results^63^. The variance in this distribution can be used to quantify the uncertainty in the prediction. Zhou et al. incorporated this technique into a 3D U-Net to obtain an uncertainty map for brain tumor segmentation. This uncertainty was then used as an additional input in a second stage to improve segmentation performance on the BraTS 2018 and 2019 dataset^64^. In test time augmentation, input images are transformed through random transforms, such as flipping, rotation, and scaling. The inference is then performed for each of the transformed views of the original input. This creates a distribution of outputs for each input image. Statistics about this distribution, such as variance end entropy can then be used to estimate uncertainty^65^. This approach has also been used for quantifying the uncertainty of brain tumor segmentation^66^.

Generalizability plays an important role in medical DL application, as trained models may be applied to datasets with different characteristics, such as disease manifestation or image acquisition techniques. This change in the dataset is commonly referred to as domain shift. Hoebel et al. demonstrated the stability of uncertainty estimates for brain tumor segmentation quality assessment even under domain shift between high- and low-grade gliomas^67^. This suggests the potential for reliable uncertainty-based quality control tools in clinical practice, although further investigation is needed to confirm generalizability across various scenarios.

### Quantification

Quantification of tumor segmentations plays an important role in the clinical applications of DL-based segmentation models. Metrics such as tumor dimensions and volume are regularly used as criteria for diagnostic and disease monitoring purposes. Examples of this include the response assessment in neuro-oncology (RANO) and the response assessment in pediatric neuro-oncology (RAPNO)^68–70^. This score is derived from 2D measurements of the tumor’s maximal diameter and is used to assess treatment response in brain tumors. An automated deep-learning approach using a U-Net-based segmentation model showed high correlation with human raters for both 2D and volumetric segmentations^71,72^.

One important challenge with manual dimension measurements is their poor inter-rater reproducibility^73^. This can potentially be solved by employing automated deep-learning-based measurements, that have shown superior repeatability and reliability compared to human readers^72^. Furthermore it has been shown that manual 2D RANO measurements are inferior compared to 3D volumetric measurements^16^. DL-based segmentation models play an important role in the clinical application of these volumetric evaluations as they provide high-quality annotations many orders of magnitude faster than a human annotator. Indeed, deep-learning-based assessment of tumor response has been shown to be a significantly better predictor of overall survival compared to RANO assessment^16^.

### Response assessment

Comparing the most recent study to prior patient imaging is a core component of a radiologist’s workflow for brain tumor assessment. It has been argued that most current DL algorithms are not suitable for applications in tasks where comparisons with previous images are necessary^74^. Developing DL algorithms that can process longitudinal imaging data, therefore, plays an important role towards advancing DL in radiology.

Kickingereder et al. developed an application-ready software infrastructure for deep-learning-based segmentation of brain tumors^16^. They utilized spatial and temporal tumor volume dynamics to predict patient time to progression. This approach included the functionality to track lesions across time points and consider new lesions. This tracking is necessary for longitudinal volume and progression monitoring based on individual lesions. Oermann et al. developed an architecture that can segment brain metastases using longitudinal imaging data^28^. The architecture, which they call “segmentation through time”, uses a collection of U-Nets. For time points after the baseline, the segmentation network also incorporates information about previous time points propagated by convolutional long short-term memory (LSTM) blocks. LSTM blocks are a type of neural network architecture that can learn and remember patterns over long sequences of data, making them well-suited for tasks involving time-series data, such as analyzing changes in medical images over multiple time points. Patel et al. developed a joint image registration and segmentation network called SPIRS to segment a new time point scan using prior time point information^75^. In their network, prior time point imaging is affinely and deformably warped onto the new time point image. The warped prior time point annotation is then used as a coarse initialization for the segmentation of the new time point. This approach significantly improves the segmentation performance for micro-metastatic brain lesions.

Another approach for estimating the treatment response of a tumor over time is through tumor growth modeling. Cell proliferation and migration within tumors can be mathematically modeled using a set of partial differential equations. However, their clinical application is hindered by the challenge of accurately estimating model parameters and other factors such as the initial tumor cell density from medical images. Recent research has explored DL-based approaches to overcome these limitations and improve parameter estimation efficiency and accuracy^76,77^. This has made it possible to construct models that are able to obtain accurate representations of tumor cell distribution and proliferation parameters from a single MRI scan. Predicting tumor invasion has important consequences for radiotherapy planning, as it would enable radiation oncologists to more accurately define radiation margins around the tumor, potentially targeting more of the tumor while reducing damage to healthy tissue^77^.

### Radiation therapy

Together with surgical resection and chemotherapy, radiation therapy is a fundamental component of treatment planning for many brain tumors. In order to maximize the effect on the tumor while minimizing adverse side effects of the radiation, the tumor and *organs at risk*, such as the brainstem, eyes and optic chiasm need to be carefully outlined on imaging in order to optimize the dose distribution. Additionally so called *barrier structures*, anatomical structures that are natural barriers to tumor spread (i.e. falx cerebri) are delineated in the imaging to optimize the margin around the actual tumor in which radiation is applied. While radiation planning is primarily performed on CT, MRI may also be acquired and used for enhanced soft-tissue contrast to aid in the delineation of the target and surrounding structures. In clinical practice these processes can be quite time-consuming, with human annotators requiring about 20 min to perform the contouring of relevant structures for a single patient^78^. Thus, there exists a clinical need for fast, human-level delineation through automation. Different deep-leaning-based solutions have been proposed for this purpose^78–81^ and the GLIS-RT open dataset is available for benchmarking model development^82^. DL-based methods have achieved excellent results in segmenting larger structures, such as tentorium cerebelli, brain sinuses, or ventricles, with clinically acceptable accuracy. Furthermore DL models have been shown to be more consistent in their outputs compared to human experts^81^. However further development is needed to provide clinically acceptable segmentations of smaller structures such as the optic apparatus^78^.

A particular challenge with radiation planning segmentation is the presence of post-treatment changes, such as resection cavities, blood products, and gliosis^83^, which are under-represented in existing datasets and segmentation models, with a recent survey finding that over 98% of published research on glioma segmentation using pre-surgical imaging^58^. The 2024 BraTS challenge, for the first time, focuses on the important task of segmentation of post-treatment brain MRIs^83^, and as such research, this area is likely to receive considerable attention in the near future.

Once the target, organs at risk and barrier structures are delineated, deep learning methods may additionally assist in selecting parameters for generating the radiation treatment plan, however to our knowledge this has only been demonstrated with CT imaging^84,85^.

## Classification

Beyond segmentation, applications of DL to brain tumor imaging can also include classification tasks. The most common architecture in medical image classification tasks is the ResNet, introduced by He et al. in 2015^86^. It first uses convolutional filters to extract important features from the image, similar to how the U-Net does for segmentation. These features are then passed through a series of layers, each containing multiple convolutional filters that identify increasingly complex patterns. Shortcut connections within these layers help preserve information and improve learning. Finally, a classification layer analyzes these extracted features and assigns the image to one of the predefined categories.

Since much of the development of DL models relies upon the availability of data, there has been less work in this area compared to segmentation, as there are fewer publicly available datasets.

### Distinguishing tumor recurrence and radiation necrosis

Distinguishing tumor recurrence from radiation necrosis presents a significant challenge in glioma management. This distinction is clinically critical as it entails fundamentally different treatment approaches. However diagnosing this accurately can be difficult even for experienced clinicians as both conditions can exhibit similar features on conventional MRI. This challenge arises from the complex and often subtle differences in tumor appearance and tissue response to radiation. In contrast, deep learning models excel at analyzing intricate patterns in imaging data. Studies have developed different CNNs that use multi-modal MRI data to distinguish between recurrence and radiation necrosis^87,88^. They show promising results, with one model significantly surpassing the accuracy of clinicians on this task^87^.

### Survival prediction

Another area in which DL promises to enhance the clinical management of brain tumors is survival prediction. There are two popular DL-based approaches to the task: a multi-class classification problem or a Cox proportional hazards model. From 2017 to 2020, the BraTS challenge included a task focused on predicting the overall survival (in days) of glioma patients who had undergone gross tumor resection from MRI data. The top-performing approach from 2020 employed a two-stage strategy. First, a segmentation model was used to delineate the tumor and its sub-compartments. Next, features derived from the number of disconnected tumor segments, along with the patient’s age, were inputted into both a linear regression model and a random forest classifier. These models were then combined, achieving an accuracy of 60% on the test dataset for classifying the patients into long-term survivors (>15 months), mid-term survivors (10–15 months), and short-term survivors (<10 months)^89^. A different study explored the use of a convolutional denoising autoencoder (DAE) network combined with a Cox proportional hazards model for survival prediction in glioblastoma patients. The DAE was used to extract features from multi-modal MRI data, which were then fed into the Cox model for survival analysis. This approach achieved a concordance index (C-index) of 0.74 on the test set^90^. Although these findings from both approaches demonstrate the potential of DL for survival prediction, achieving the level of accuracy required for robust clinical implementation remains an open challenge.

### Biomarker prediction

One of the most promising applications of DL in brain tumor analysis is the prediction of genetic biomarkers directly from imaging data. This idea carries transformative potential for clinical practice, as it could enable clinicians to obtain important information about a tumor’s genetic profile without the need for invasive biopsies or surgeries.

One example is the prediction of isocitrate dehydrogenase (IDH) mutation status in gliomas. IDH mutation status is an important factor in determining the prognosis and treatment of gliomas, and being able to identify it pre-treatment can significantly impact clinical decision-making. Deep learning-based methods have shown promise in predicting IDH status from MRI scans, offering a non-invasive alternative to traditional biopsy-based methods^9,15^. A model that first performed automated tumor segmentation and subsequently used both radiomics and deep-learning derived features was able to predict the IDH mutational status of patients diagnosed with gliomas with an accuracy of 78.8% and 93.8% on internal and external test sets, respectively. This model used three different MRI sequences (T1 post-contrast, T2 and FLAIR) as its input^15^. A recent publication explored the ability of a deep learning model to predict the mutational status of the BRAF gene in patients with pediatric low-grade gliomas based on T2-weighted MRI scans. The model was able to classify BRAF status into three classes (BRAF fusion, BRAF V600E, and wild-type) with an accuracy of 75% and 77% on internal and external test sets, respectively^91^.

Calabrese et al. implemented an approach in which the tumor sub-compartments (i.e. enhancing tumor, non-enhancing tumor, and edema) were first segmented by a deep-learning model and subsequently used as the basis for radiomics feature extraction. These features were then passed to a random forest regression model. The authors explored the predictive capabilities of the model for nine different genetic biomarkers in glioblastoma. The model showed good results for predicting IDH mutations, ATRX mutations, chromosome 7/10 aneuploidies, and CDKN2 family mutations. The sensitivity for those biomarkers ranged from 0.76 to 0.94 and the specificity from 0.86 to 0.92^92^.

A widely discussed use-case is the prediction of O6-methylguanine-DNA methyltransferase (MGMT) promoter methylation status, which is a key indicator of response to temozolomide chemotherapy in glioblastoma. There is a large public dataset created for the BraTS 2021 challenge, which provides information on the MGMT promoter methylation status along with MRI scans for 2040 patients^93^. However, the feasibility of accurately predicting MGMT status from MRI data remains controversial. While some studies have reported promising results^94–96^, others have questioned the validity of these findings and argued that predicting MGMT status from MRI alone may not be possible with current techniques^97,98^. This highlights the importance of critically examining results and ensuring transparency in DL research, especially when considering clinical applications. Notably, in the 2021 BraTS challenge, the winning model for MGMT prediction achieved an AUROC (area under the receiver operating characteristic curve) of only 0.62, which is considered poor and certainly not sufficient for reliable clinical decision-making^99^. Such examples underscore the need for rigorous validation and cautious interpretation of DL models before integrating them into clinical workflows. Overall, the use of DL to predict genetic biomarkers from imaging data is a rapidly evolving field with significant potential to improve the diagnosis and treatment of brain tumors.

## Future directions

While DL has demonstrated remarkable progress in brain tumor analysis, the field continues to evolve rapidly, with several promising avenues for future development.

### Quantitative MRI

Quantitative MRI methods^100,101^, while not widely deployed clinically, hold promise to increase the standardization of images between vendors by directly quantifying tissue properties, though other qualitative variations are likely to persist. Further, early evidence suggests that such images may provide further insight into tumor characteristics, such as infiltration beyond the contrast-enhancing region visible in conventional qualitative images^102^. However, currently, there is a lack of experimental studies demonstrating the value of quantitative MRI for developing AI models. For example, Tampu et al.^103^ found no statistically significant advantage of utilizing quantitative relaxometry images over conventional T1 and T2 weighted images for developing AI models for the detection and identification of brain tumor biomarkers, however, the study was conducted on a very small dataset of 23 patients. Future work should focus on investigating the value of quantitative MRI for AI model development across larger datasets and multiple vendors.

### Multimodal integration

The radiographic appearance of a tumor in an MR image cannot capture the full complexity of a brain tumor in its full clinical context. Consideration of other clinical information, including patient demographics, genomics, and histopathology is therefore crucial for clinical decision-making. However, despite the fact that deep learning models can naturally integrate many high-dimensional data types, most current work considers only a single modality. DL has already shown promise in analyzing other data types from brain tumor patients. For instance, deep learning models trained on whole-slide images (WSI) of histopathology slides have achieved high accuracy in predicting 1p/19q codeletion status in gliomas, surpassing the performance of traditional methods like fluorescence in situ hybridization (FISH)^104^. Initial studies have explored the integration of WSI data with genomic and transcriptomic information to predict survival outcomes in glioma patients^105^, but there remains considerable potential for improved predictions by leveraging multi-modal models.

### Vision transformers

A recent and important development is the transformer architecture^106^. While initially developed for applications related to natural language processing, it was adapted towards image-based tasks by Dosovitskiy et al.^107^. Due to the more flexible design of their proposed vision transformer (ViT) architecture, it is better able to capture long-range interactions within an image, meaning it can understand relationships between distant parts of the image, which is important for tasks like identifying complex shapes or patterns. As such, ViTs have been shown to outperform CNNs when given sufficiently large amounts of training data^107^. Since most brain tumor datasets are small, the potential benefits are yet to be realized. However, as the availability of large dataset sizes improves, ViTs may become increasingly used for brain tumor image analysis.

### Foundation models

Another promising future direction is the development of foundation models for brain tumor imaging. Foundation models are an emerging paradigm in DL that involves the self-supervised training of large, general-purpose models on very large datasets of diverse data types^108^. These models learn fundamental patterns and relationships within the data, enabling them to perform a wide range of downstream tasks with remarkable accuracy and efficiency. Unlike traditional DL models that are trained for specific tasks, foundation models are adaptable and can be fine-tuned for different applications without requiring extensive retraining. Chen et al. recently introduced a general-purpose foundation model for pathology that was pretrained on more than 100 million images acquired from over 100,000 diagnostic H&E-stained WSIs across 20 different tissue types^109^. This model, after fine-tuning with limited task-specific data, achieved excellent performance on a range of tasks, including brain metastasis detection, glioma IDH1 mutation prediction, and histomolecular subtyping. The model showed excellent performance even on few-shot tasks for which only between 1 and 32 task-specific training examples per class were provided to the model. This is orders of magnitude fewer examples than would be needed without a foundation model. Another recent publication proposed a foundation model for cancer imaging biomarker discovery using computed tomography (CT) data from over 11,000 radiographic lesions^110^. After fine-tuning with limited data, their model outperformed other state-of-the-art models on a variety of tasks such as predicting malignancy in lung nodules and predicting survival in non-small cell lung cancer (NSCLC). Although their analysis did not include brain lesions or MRI images, the results demonstrate the potential of similar foundation models for MRI in neuro-oncology applications where large datasets are not available. The adoption of such foundation models is likely to accelerate future research on brain tumor analysis.

## Limitations and challenges

Despite the significant progress over recent years in the application of DL to brain tumor analysis, only a small number of brain tumor-related models are approved for clinical use within the United States^111^. There remain substantial challenges to further research progress and its translation.

### Datasets

It remains challenging to collate imaging datasets for applications beyond those covered by the existing public datasets^112^. Though vitally important, patient privacy and consent are the most important barriers to the widespread sharing of medical imaging data outside of individual hospitals and radiology providers, which individually see relatively small numbers of patients compared to those needed to train accurate deep learning models. In addition to the images themselves, curating or creating appropriate and accurate ground truth presents a further challenge, as the process is typically time-consuming and requires considerable expertise, with 3D segmentations, in particular, being slow to generate. Where manual processes are involved, there is often considerable inter-reader variability, which may or may not be clinically significant depending on context. Previous studies have found substantial variation between readers in obtaining quantitative measurements manually from MRIs^113–115^. Even when large datasets are created and released publicly, the utility of those images is limited by the availability of accompanying ground truth. For example a dataset of segmented brain MRIs containing tumors cannot be used for a study on outcomes prediction if no information on outcomes was released.

As a consequence, most studies in brain tumor analysis either focus on one of the existing public datasets, leading to the over-representation of three associated tasks in the literature, or use small, often single-institutional datasets that lack diversity. In particular, the availability of large datasets has led to a focus on gliomas, brain metastases, and meningiomas at the expense of work relating to rarer brain tumors, such as craniopharyngiomas, pineoblastomas, or parameningeal rhabdomyosarcomas.

Major initiatives such as the US National Cancer Institute’s Imaging Data Commons (IDC)^116^ and the European Federation for Cancer Images (EUCAIM), as well as challenges, such as BraTS will continue to serve an important role in collating and standardizing access to imaging data at scale for the research community. Furthermore, federated learning^117,118^, a technique wherein models are trained across multiple sites without data leaving each site, will likely play an increasing role in model development in order to create large datasets while retaining patient privacy but it is not without its own technological and logistical challenges.

### Reproducibility of research

The high variability of brain MRI data coupled with the small number of cases often used in (frequently private) datasets leads to this area being particularly vulnerable to issues surrounding lack of reproducibility. In many cases, published articles have not yet been replicated by further studies, and in some cases further studies have been conducted but failed to replicate the previous findings^97^. Results obtained on small, private datasets, should be treated with caution as they fail to generalize beyond a single institution or MRI scanner and may be the result of unintended bias or spurious correlation present in the training data (for example, between a clinical outcome and the scanner used to acquire the image), or may be the result of chance. In some cases methodological failures may have given rise to reporting of incorrect results: as an example, a paper on the prediction of MGMT status from gliomas was withdrawn upon the discovery of an error in the computer code used to conduct the study^119^, and a review of machine learning in radiomic analyses (not using DL) identified common mistakes that inflate performance^120^.

Where possible, the public release of datasets and source code can help to reduce the likelihood of replication issues in research.

### Domain shift and generalizability

As noted above, DL models can fail to generalize beyond the sites and scanners represented within their training data, which creates a considerable challenge for deploying DL at scale, and the flexibility and complexity of MRI as a modality make this a particular concern for brain tumor analysis^46^. In addition to changes between sites, changes over time at a single site are due to factors including scanner software, imaging protocols, clinical workflows, and patient demographics, and this, in turn, can lead to performance degradation^121,122^.

### Bias and fairness

Another crucial consideration is fairness. While there is no single definition of fairness within AI, generally speaking, it refers to unequal model performance on different subpopulations, for example, different races, genders, or ages^123,124^. Unequal model performance can, in turn, lead to unequal outcomes in clinical care. The causes of unfairness may simply be under-representation of subpopulations within the training data, but it can also be more insidious and difficult to avoid. Biases and outcome differences that exist within healthcare—and therefore in model training data —can be propagated by artificial intelligence models^125,126^, because models can learn to infer protected characteristics, such as age, race, and gender even if they not provided directly to a DL model^127^, and then learn to associate these with, for example, poorer outcomes that are a result of socio-economic factors.

Therefore, model developers, especially those developing models intended for clinical use, should, therefore, follow established guidelines to screen their models for fairness across any relevant subpopulations^123,124^.

### Clinical translation

Although deep learning models have significant potential to benefit patient care, incorrect predictions or inappropriate use of DL models pose a significant risk of harm. Several steps are crucial to minimize harm^128^, including thorough validation of models on representative data for clinical effectiveness and education of physicians in the capabilities and limitations of the technology^129^ to reduce the risk of automation bias, where physicians blindly follow the predictions of an algorithm^130^.

### Explainability

Where DL models are to be used for cancer treatment decision-making, it is vital that their predictions are understandable to clinicians. Unfortunately, most DL models, including most articles in this review, provide black-box predictions. Building interpretable models remains one of the major unsolved technical challenges within the field of DL. Many approaches to the explainability of DL methods rely on determining which regions of the image are relevant to the prediction using techniques, such as saliency or occlusion maps. However, this level of explanation is likely to be insufficient for most of the applications discussed above. Counterfactual explanations, which allow the user to visualize how the image would need to change to change the prediction, may provide one more promising direction^131^.
